# Effectiveness of cognitive training on Lamb-Shaffer syndrome: a case report

**DOI:** 10.1590/1980-5764-DN-2026-0524

**Published:** 2026-08-24

**Authors:** Gabriel Henrique Velozo Gonçalves, Fernanda Buzzinari Ribeiro de Sá, Nadia Shigaeff

**Affiliations:** 1 Universidade Federal de Minas Gerais, Faculdade de Filosofia e Ciências Humanas, Programa de Pós-Graduação em Psicologia: Cognição e Comportamento, Belo Horizonte MG, Brazil.; 2 Universidade Federal de Juiz de Fora, Hospital Universitário, Juiz de Fora MG, Brazil.; 3 Universidade Federal de Juiz de Fora, Instituto de Ciências Humanas, Departamento de Psicologia, Juiz de Fora MG, Brazil.

**Keywords:** Case Reports, Rare Diseases, SOXD Transcription Factors, Cognitive Training, Relatos de Casos, Doenças Raras, Fatores de Transcrição SOXD, Treino Cognitivo

## Abstract

Lamb-Shaffer Syndrome (LAMSHF) is a rare neurodevelopmental condition caused by haploinsufficiency of the SOX5 gene, characterized by intellectual disability, language impairments, and executive dysfunctions. Few studies have explored therapeutic interventions for this population. This case report describes the cognitive profile and intervention outcomes of a 20-year-old woman diagnosed with LAMSHF. The patient presented with difficulties in attention, memory, language comprehension, and executive functions. A neuropsychological assessment confirmed mild intellectual disability and specific deficits in working memory, attention, and verbal memory. A cognitive training program was conducted targeting working memory and attention. Post-intervention reassessment revealed improvements in working memory, alternating and divided attention, and verbal long-term memory. These findings suggest that non-pharmacological cognitive training may provide improvements in cognitive functioning for individuals with LAMSHF. Given the scarcity of evidence-based treatments for rare neurodevelopmental syndromes, cognitive training may represent a promising therapeutic alternative.

## RESUMO

A síndrome de Lamb-Shaffer (LAMSHF) é uma condição do neurodesenvolvimento rara causada pela haploinsuficiência do gene SOX5, caracterizada por deficiência intelectual, dificuldades de linguagem e disfunções executivas. Poucos estudos exploraram intervenções terapêuticas para essa população. Este relato de caso descreve o perfil cognitivo e os resultados da intervenção de uma mulher de 20 anos diagnosticada com LAMSHF. A paciente apresentava dificuldades de atenção, memória, compreensão da linguagem e funções executivas. Uma avaliação neuropsicológica confirmou deficiência intelectual leve e déficits específicos na memória de trabalho, atenção e memória verbal. Foi realizado um programa de treinamento cognitivo voltado para a memória de trabalho e a atenção. A reavaliação pós-intervenção revelou melhorias na memória operacional de curto prazo, atenção alternada e dividida e memória verbal de longo prazo. Estes achados sugerem que o tratamento não farmacológico por meio de treinamento cognitivo pode proporcionar melhorias no funcionamento cognitivo de indivíduos com LAMSHF. Dada a escassez de tratamentos baseados em evidências para síndromes neurodesenvolvimentais raras, o treinamento cognitivo pode representar uma alternativa terapêutica promissora.

## INTRODUCTION

Rare diseases are defined as those affecting 65 individuals per 100 thousand people^1^. Around 40% of them have a genetic cause^2^, normally accompanied by degenerative and debilitating nature, causing significant difficulties on the daily life of diagnosed individuals, and most often not having a treatment oriented to curing the disease^3^, but only to providing lifelong support.

Lamb-Shaffer Syndrome (LAMSHF) is considered a rare disease, specifically a neurodevelopmental syndrome. It is caused by haploinsufficiency of the SOX5 gene, which can be a spontaneous or inherited mutation^4,5^. This gene encodes a transcription factor that is involved in the regulation of embryonic development and consequent development of the central nervous system, helping to regulate cell fate, neurogenesis and chondrogenesis, and can also affect the development of other body systems^6,7^. Diagnosis of the syndrome involves the use of a genetic test, such as array comparative genomic hybridization (aCGH/CGH-Array)^4^ or EXOMA, based on the identification of signs of the syndrome. Because it is a recently discovered rare syndrome, there is no clear epidemiological data available.

This report describes the case of a woman with the diagnosis of LAMSHF, focusing on the cognitive aspects of the syndrome.

## CASE PRESENTATION

We report the case of a 20-year-old woman, with 12 years of education, diagnosed with LAMSHF in 2018, by aCGH, with a microdeletion on chromosome 12 (12. p12.1), with no available biological family history of similar syndromes, with a history of premature birth at seven months’ gestation, and a history of alcoholism during pregnancy. She also had the diagnosis of labyrinthitis and bilateral keratoconus, both under medical supervision and not directly related to LAMSHF in previous literature. A support teacher was needed during schooling. At the time the intervention was conducted, the participant was being monitored by a psychiatrist and psychologist to treat anxiety, and also being annually monitored by a geneticist. She had already been monitored by a speech therapist and an occupational therapist, with improved functionality.

The initial complaints included maintaining concentration, with episodes of inattention and disorientation, changes in working memory and language difficulties, especially comprehension. These complaints were corroborated by the participant’s adoptive mother.

At first, the anamnesis showed physical and cognitive symptoms. The physical ones included increased sensitivity to smells, tension headache at the front of the head, associated with dizziness and nausea — these symptoms correlated with the labyrinthitis — and scoliosis. Tinnitus in the ears, occasional low-intensity tremors in the lips and visual impairment due to bilateral keratoconus, with the use of corrective lenses, were also highlighted. It should be noted that the participant had undergone two surgeries in childhood, a hernia and an unspecified nose surgery.

With regard to cognitive symptoms, the anamnesis reported: difficulty with mental flexibility; reduced speed of thought; complaints concerning language, such as understanding what is being said by other people or what is being read; high distractibility, with loss of reasoning and difficulty finding the right words; forgetting what she was doing, where objects were left, and occasionally forgetting the order of events; difficulty with mathematical operations, including everyday ones; problems with “predicting” time, such as understanding how much time is left until a certain appointment. These symptoms are reported to have existed since childhood, but are more markedly observed from school age onwards and with greater demands on daily life.

After the anamnesis, a neuropsychological assessment was conducted to delve further into the complaints. The instruments used were the Mini-Mental State Examination (MMSE), Hopkins Verbal Learning Test — Revised (HVLT-R), Boston Naming Test (BNT), the subscales Digit Span, Vocabulary, Comprehension and Matrix Reasoning from WAIS-III, the Color Trails Test, the Victoria Stroop Test, the Beck Anxiety Inventory and the Beck Depression Inventory. Also, when necessary, cognitive tasks related to a domain were used when there was no available neuropsychological test for the examination.

The MMSE showed a score of 24, indicating cognitive impairment, considering the participant’s level of education. This result was better assessed in depth in specific cognitive domains, namely: general intelligence, working memory, short- term and long-term memory, language, executive functions, mood and functionality. The main result was a mild intellectual disability. Although the existence of intellectual disability may explain the cognitive symptoms, some results showed significantly increased impairment in the participant’s baseline performance, including in working memory, immediate short-term and episodic long-term memory (verbal), changes in language comprehension, and generalized impairment in attention, with greater severity in divided, alternating and selective attention. Mild to moderate mood impairment was also highlighted as a potential confounding factor detrimental to cognitive performance. Despite the altered results in language comprehension, fluency and naming ability were preserved, as was writing ability, although a slight reduction in vocabulary and occasional grammatical errors were observed, most of which were self-corrected. There was no impairment in performing basic activities of daily living.

### Therapeutic intervention

#### Cognitive training

A cognitive training was carried out with two main cognitive domains: attention, with a special focus on sustained and alternating attention, and executive functions, notably working memory, as these were the most impactful results in the anamnesis and assessment and potential bases for other deficits. The training took place over a total of nine sessions, five of which focused on executive functions and three on attention, as well as a feedback session.

Each cognitive training session included an initial psychoeducation in which an explanation was given about the cognitive domain to be worked on, followed by one or two tasks to train this domain, and finally an explanation of the results of the tasks, and a “homework assignment” to be completed by the next session. Each session lasted around 60 minutes.

After the cognitive training, a neuropsychological reassessment was carried out post-intervention, to compare with the results of the pre-intervention assessment and identify whether the cognitive training had brought benefits to the participant. A debriefing of the results took place after the entire process.

During the nine cognitive training sessions, the participant was collaborative in carrying out the interventions, with slight variations in interest and tiredness, which were taken into account in each session. In the first two sessions, it was necessary to adapt the difficulty of the tasks, starting with simpler levels than expected. From the third session onwards, there was a progressive improvement, which made it possible to increase the difficulty in the following sessions, especially the later ones. Session 5 was a feedback session on the progress of the training. Table 1 summarizes the rationale of the training and the tasks used.

**Table 1.** Cognitive domains trained, tasks used and brief description of the tasks.

Session 1		
Cognitive domain assessed	Task	Task description
Executive Functions (Working Memory)	Ordering months	The participant must sort the months of the year in ascending order, the months being spoken by the evaluator. E.g.: spoken years “December, May, August, April” must be spoken back as “April, May, August, December”
**Session 2**		
Cognitive domain assessed	Task	Task description
Executive Functions (Working Memory)	Organizing the story	The participant must read a story and, after a period of time, write down everything they remember about it.
	Sequence of numbers	The participant must repeat a sequence of numbers spoken by the evaluator; the sequence of numbers varies in complexity, requiring new manipulations as the patient progresses, such as the amount of numbers, or speaking in an inverted order.
**Session 3**		
Cognitive domain assessed	Task	Task description
Executive Functions (Working Memory) and Sustained Attention	Mark the target object	The participant will be presented with target stimuli. Next, they must find these target stimuli within a cluster of distractor stimuli, with varying amounts of time to do it, while keeping the target stimuli in memory.
	Supermarket items	The participant must memorize a number of items from a list of items commonly found in supermarkets, and after a period of time they must write down as many items as they can remember, with memorization strategies being taught, such as arranging items according to the aisles and shelves in a supermarket.
**Session 4**		
Cognitive domain assessed	Task	Task description
Executive Functions (Working Memory)	Sequence of numbers	The participant must repeat a sequence of numbers spoken by the evaluator; the sequence of numbers varies in complexity, requiring new manipulations as the patient progresses, such as the amount of numbers, or speaking in an inverted order.
	Ordering months	The participant must sort the months of the year in ascending order, with the months spoken by the assessor. E.g.: spoken years “December, May, August, April” must be spoken back as “April, May, August, December”.
**Session 5**		
Cognitive domain assessed	Task	Task description
Feedback	-	The participant is asked to give feedback on how the sessions have been going, any difficulties encountered, and whether there have perceived any changes in daily life.
**Session 6**		
Cognitive domain assessed	Task	Task description
Executive Functions (Working Memory) and Sustained Attention	Mark the target object	The participant will be presented with target stimuli. Next, the patient must find these target stimuli within a cluster of distractor stimuli, with time variations to find it, while keeping the target stimuli in memory.
	Mazes	The participant must sustain attention during the task in order to successfully complete each maze, in succession and with increasing difficulty, and afterwards must repeat the mazes in order to reduce the completion time.
**Session 7**		
Cognitive domain assessed	Task	Task description
Sustained Attention	Mazes	The participant must sustain attention during the task in order to successfully complete each maze, in succession and with increasing difficulty, and afterwards must repeat the mazes in order to reduce the completion time.
**Session 8**		
Cognitive domain assessed	Task	Task description
Sustained and Alternating Attention	Make the trail	The participant must connect numbers in ascending order, alternating the colors of the circles in which the numbers are inserted.
	Mark the target object	The participant will be presented with target stimuli. Next, they must find these target stimuli within a cluster of distractor stimuli, with time variations to find it, while keeping the target stimuli in memory.
**Session 9**		
Cognitive domain assessed	Task	Task description
Alternating Attention	Switching between verbal stimuli	The participant must, together with the researcher and one volunteer — in this case, a family member of the patient — switch between stimuli, speaking them aloud, based on predetermined categories. The inclusion of additional people was necessary due to the nature of the task. E.g.: P1 — “one”, P2 — “Amanda”, P3 — “two”, P1 — “Bruno”.
	Make the trail	The participant must connect numbers in ascending order, alternating the colors of the circles in which the numbers are inserted.

#### Neuropsychological reassessment

In the neuropsychological reassessment, the cognitive tests and tasks applied were the same as in the initial assessment. The MMSE showed a score of 27, a slight improvement on the previous assessment. Compared to the previous assessment, and considering the training focuses, gains were observed in working memory, with the span increasing from 2 to 5, meaning that the participant increased the number of elements she was able to hold in memory. Furthermore, there was also an improvement in alternating attention performance, with the initial assessment result being observed as “severely altered”, and after the intervention it fell within the normal range, i. e. she showed preserved performance.

There was also an improvement in other cognitive domains not directly trained, such as a significant gain in divided attention, a significant improvement in immediate long-term memory for verbal material and in the ability to recognize verbal material, as well as a slight improvement in language comprehension skills. There was also a significant reduction in mood impairment. A slight worsening in vocabulary was also found. Finally, despite the objective gains observed, the assessment of general intelligence remained stable. Table 2 shows the results of each test for pre- and post intervention.

**Table 2.** Neuropsychological test results of pre- and post-intervention.

Neuropsychological test	Pre-intervention	Post-intervention
Mini-Mental State Examination	24/30*	27/30*
Boston Naming Test (Language)	-0.6+	-0.33+
Vocabulary (Language)	Weighted Score: 7§	Weighted Score: 6§
Matrix Reasoning (Visual Processing/Fluid Intelligence)	Weighted Score: 8§	Weighted Score: 8§
Digit Span (Working Memory)	Weighted Score: 4§ Total Digits Forward: -2.45+ Total Digits Backward:-1.76+	Weighted Score: 6§ Total Digits Forward: -2.45+ Total Digits Backward: 0.53+
Comprehension (Verbal Reasoning)	Weighted Score: 5§	Weighted Score: 7§
Hopkins Verbal Learning Test - Revised (HVLT-R) (Memory)	Immediate: -4.06+ Delayed: -3.35+ Recognition: -1.98+	Immediate: -0.62+ Delayed: -2.73+ Recognition: 0.54+
Estimated IQ	81	79
Color Trails Test (Sustained and Divided Attention)	Trail 1: -0.45+ Trail 2: -2.05+	Trail 1: -0.42+ Trail 2: -0.64+
Stroop Test (Selective Attention)	Card 1: -2.25+ Card 2: -2.27+ Card 3: -5.89+	Card 1: -1.44+ Card 2: -0.52+ Card 3: -3.93+
Beck Depression Index	14/63*	2/63*
Beck Anxiety Index	19/63*	15/63*

Notes: *Total points as per correction from instruction manual of the neuropsychological test;

+results as per Z-score;

§weighted score.

Throughout the sessions, qualitative improvements were observed, such as better understanding of the instructions given, as well as a decrease in the total time taken to complete each activity. The participant also reported an improvement in her quality of life, with improved attention span, greater comprehension of the content read, greater ability to retain information in memory and to deal with more than one stimulus at the same time.

## DISCUSSION

This case report aimed to assess whether cognitive training can serve as a treatment for the cognitive symptoms of LAMSHF. The results indicate that training can bring considerable benefits to some of the deficits presented, especially attention and working memory.

There was an increase in the results of MMSE, even though it was still considered as below the expected average, considering the patient’s schooling. The results for tests assessing the language as a cognitive domain, including Comprehension, also showed an improvement, except for Vocabulary, which may have been influenced by the tiredness/disinterest observed in the patient during the post-training application. As is to be expected in patients with intellectual disabilities, the estimated general intelligence did not change significantly, even though the result post-intervention is slightly lower, influenced by the result of Vocabulary.

The participant’s subjective perception of her own abilities related to memory and attention was objectively observed by the results of the tests that measured them, which showed the biggest increases, shedding a positive light on the beneficial effects of this cognitive training.

As an unexpected result, the mood symptoms diminished, specially for depression, even though no specific task or training was created to address these. We believe this may be due to the positive changes that the training made to the participant’s life, creating a better sense of self-esteem and self-efficacy, given that they allowed her to be more independent and more understandable of her condition.

Due to the novelty of the syndrome, literature has not yet delved deep onto possibilities for treatment or cognitive and emotional support for LAMSHF. Only one study^8^ has carried out a non-pharmacological intervention focusing on cognitive symptoms of a patient with LAMSHF, mainly in the language domain, showing positive results in the ability to associate speech with concrete objects, generalization of skills for everyday life and general language comprehension. However, given the differences in the domains trained between the study by Sajewicz-Radtke et al. and the present study, the task of comparing results and systematizing a treatment program becomes arduous.

Considering the fact that the majority of neurodevelopmental syndromes do not have a pharmacological treatment, approaching these conditions with non-pharmacological procedures may be the best option to provide some type of improvement for symptoms, being an accessible therapeutic intervention. Syndromes such as Prader-Willi exhibit some cognitive symptoms similar to LAMSHF, such as language problems, global intellectual deficit and executive deficits^9, 10, 11^, as does Noonan syndrome^12, 13, 14^, and cognitive interventions have shown benefits in the cognitive abilities worked on in both^15,16^, as well as other conditions such as Fragile X syndrome^17^ and autistic spectrum disorder comorbid with intellectual disability^18^. In addition, a scoping review^19^ presented positive results on the potential usefulness of cognitive training for several other neurodevelopmental syndromes. However, as with LAMSHF syndrome, the scarcity of studies, the high methodological variability, and the lack of standardization in the interventions make it difficult to generalize and create a robust body of evidence. Therefore, more studies are needed to understand the effectiveness of cognitive training for this population, as well as a greater consensus in the literature on how these cognitive interventions should be designed in order to be scientifically based^20^.

Nevertheless, the findings of this case present an opportunity to develop functional interventions for LAMSHF, focusing on the idea that cognitive abilities can be altered. This is relevant in that, although those affected by LAMSHF suffer an enormous influence from biology and genetics, training and environment enrichment could bring them benefits.

## CONCLUSION

Rare diseases require intersectoral efforts by health systems to reduce the burden both on the family and, financially, on the country’s budget. Given the specific nature of these diseases and the lack of effective pharmacological interventions to remedy or alleviate the problem, the use of non-pharmacological strategies such as cognitive training opens up a space for new forms of treatment, with the potential to improve these patients’ quality of life and, consequently, their functionality. Thus, this work adds to an existing field of study for the production of compensatory and health promotion strategies, giving professionals new strategies in the comprehensive care of patients with rare diseases. This work also provides insights into a neurodevelopmental disorder that is yet to be fully comprehended.

## Data Availability

The datasets generated and/or analyzed during the current study are not publicly available due to ethical restrictions but are available from the corresponding author upon reasonable request.
